# Preparation of Biodiesel from Soybean Catalyzed by Basic Ionic Liquids [Hnmm]OH

**DOI:** 10.3390/ma7128012

**Published:** 2014-12-11

**Authors:** Qinggong Ren, Tongmei Zuo, Jingjing Pan, Changle Chen, Weimin Li

**Affiliations:** 1 School of Pertrochemical Engineering, Changzhou University, Changzhou 213164, China; E-Mails: qgren@cczu.edu.cn (Q.R.); zuotongmei@honglu.net (T.Z.); pjj19901120@126.com (J.P.); 2 Chinese Academy of Science CAS Key Laboratory of Soft Matter Chemistry, Department of Polymer Science and Engineering, University of Science and Technology of China, Hefei 230026, China

**Keywords:** ionic liquid, transesterification, biodiesel, catalytic

## Abstract

A morpholine alkaline basic ionic liquid (IL) 1-butyl-3-methyl morpholine hydroxide ([Hnmm]OH) was synthesized and characterized by ^1^H NMR and FT-IR. [Hnmm]OH is highly active in catalyzing the synthesis of biodiesel from the reaction of methanol with soybean oil. The influence of the reaction conditions, including the [Hnmm]OH catalyst amount, the molar ratio of methanol to soybean oil, reaction temperature and time, was investigated. Moreover, the pH and thermal stability of the catalyst was studied. The catalytic activity was affected by its alkalinity. The optimum reaction conditions were found as [Hnmm]OH amount of 4% (mass fraction), the methanol to soybean oil molar ratio of 8, temperature 70 °C and reaction time 1.5 h, the yield of Biodiesel reached 97.0%, and exhibits high stability upon recycling, the yield of Biodiesel is still more than 90% even after being reused for five times. A great advantage of using ILs is that it is very easy to separate the final products. After the reaction, a biphasic system was obtained. The top phase contains biodiesel and a little bit of methanol. Pure biodiesel can be isolated by vacuum evacuating the methanol. The bottom phase contains methanol, glycerol and ILs. Pure glycerol can be obtained simply by distillation. After distillation, pure ILs was obtained, which can be used directly for another reaction. The as prepared biodiesel shows very appealing properties.

## 1. Introduction

With the concerns over fossil fuels shortage, crude oil price increase and vehicle emissions, it is increasingly necessary to explore alternative clean and renewable energy sources. Biodiesel, consisting of fatty acid methyl esters (FAME), as a promising non-toxic, biodegradable alternative fuel, has attracted significant attentions due to its unique and superior features. Biodiesel is becoming more popular within the European Union (EU), which has set an objective to reach 20% share of total motor fuel consumption with biodiesel by 2020 [1,2,3].

Biodiesel is produced by transesterification of triglycerides to afford lower molecular weight fatty acid monoalkyl esters and glycerol using vegetable oil or animal fat with methanol or alcohol in the presence of an appropriate catalyst [4,5,6]. The synthetic strategies include physical, chemical and enzymatic methods. Physical method is simple and generates biodiesel with low viscosity. However, the octane number is not high and the coke and oil pollution is difficult to resolve. In the enzymatic method, methanol and products can easily attach to the surface of enzyme, causing enzyme deactivation. Transeterification reaction is the main strategy used in chemical method, which is usually catalyzed by inorganic acids such as sulfuric acid and alkali (KOH and NaOH). However, there are many disadvantages including low reaction rate, low activity, low stability, high temperature, long reaction time, moisture sensitivity of the catalyst, high cost, saponification, and the difficulties in down dream recovery and purification of the product [7,8,9,10]. Therefore, it is highly desired to develop more efficient and environmentally friendly synthetic strategy.

Ionic liquids (ILs) are mostly organic salts with melting points often near or below room temperature, which is obtained from the combination of a large cation with a small anion, also known as low-temperature molten salt [11,12,13,14]. Recently, ILs have received much interest from various fields such as catalysis, separation, synthesis and electrochemistry because of its excellent properties such as negligible vapor pressure, low toxicity, high catalytic activity, excellent chemical and thermal stability, high conductivity, strong dissolution ability, potential recoverability, design possibilities [15,16,17,18]. As a result, large amounts of ionic liquids have been applied in chemical synthesis, in which the ionic liquids are employed both as solvents and as catalysts. However, there have been very few reports on the synthesis of biodiesel catalyzed by alkaline ionic liquid [19,20,21]. For example, Li *et al.* [22]. synthesized alkaline ionic liquids [Bmim]OH, and studied its catalytic effect in the reaction of the synthesis of biodiesel.

In this work, we explored the synthesis of morpholine alkaline ionic liquids [Hnmm]OH, which was characterized by ^1^H-NMR spectroscopy and FT-IR. Furthermore, its basicity and catalytic properties were investigated.

## 2. Results and Discussion

### 2.1. Catalyst Characterization

**Figure 1:**
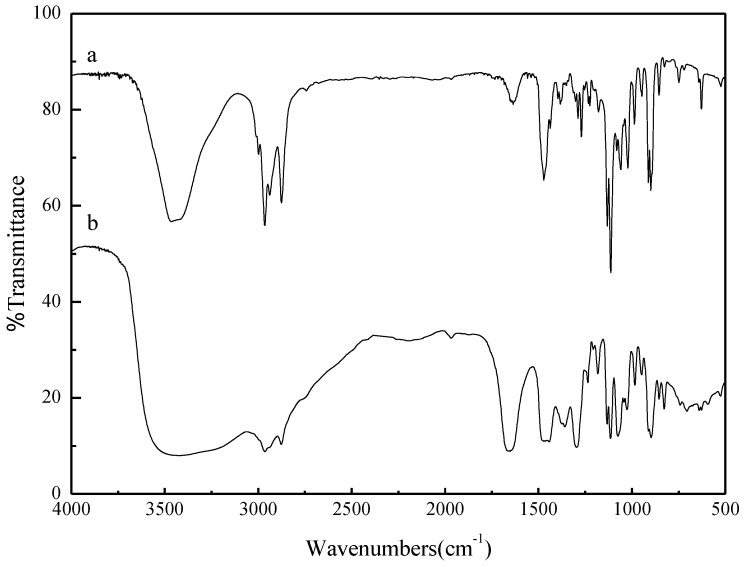
FT-IR of ionic liquids intermediates [Hnmm]Br and ionic liquids [Hnmm]OH. (**a**) [Hnmm]Br; (**b**) [Hnmm]OH.

As shown in Figure 1, [Hnmm]Br and ionic liquid [Hnmm]OH have the same cation, the peak at 3418 cm^−1^ is characteristics of the stretching vibration of –OH. The peaks at 2877.0 cm^−1^ and 2964.4 cm^−1^ are assigned as the saturated C–H stretching vibration. The peaks at 1442.6 cm^−1^ and 1651.5 cm^−1^ are assigned to the stretching vibration of C–N. The peaks at 1359.4 cm^−1^ and 1296.3 cm^−1^ are characteristics of the symmetrical deformation vibration of –CH_3_. The peaks at 1114.4 cm^−1^ and 1133.0 cm^−1^ and 1182.2 cm^−1^ are characteristics of the stretching vibration of C–O–C. Compared with [Hnmm]OH, the peak at 3465.1 cm^−1^ is strong and sharp, which is caused by intermolecular hydrogen bonding of Br–H.

### 2.2. The Basicity and the Thermal Stability of the Ionic Liquids

The basicity of different concentration of IL in water was determined by Delta320PH (Mettler Toledo, Columbus, OH, USA) defined by PH. The catalytic properties of ionic liquids were affected directly by its concentration, and correspondingly the basicity in water. As shown in Table 1, the basicity of ionic was less than KOH under the same concentration. However, as a big advantage of the ionic liquids, it could be well dissolved in common organic solvent, and serves as catalyst and solvents at the same time. Therefore, the homogeneous condition is maintained.

**Table 1:** Alkalinity of [Hnmm]OH.

Solution concentration (g·mL^−1^)	[Hnmm]OH, pH	KOH, pH
0.005	11.981	12.970
0.01	12.350	13.244
0.05	13.044	13.900
0.1	13.336	14.196

**Figure 2:**
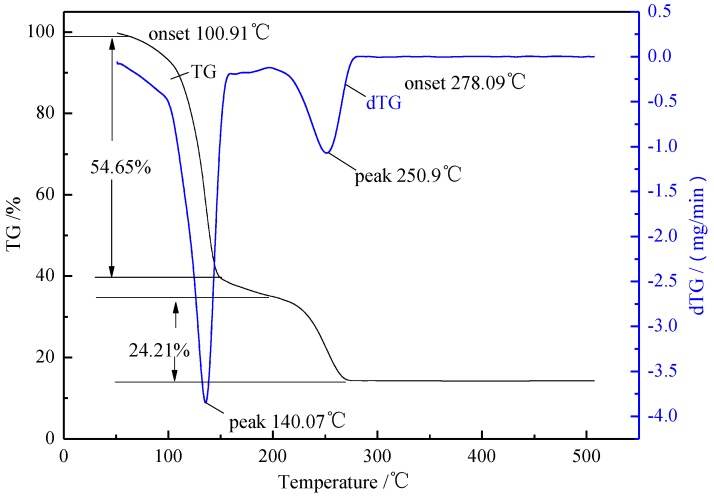
TG analysis of various ionic liquids [Hnmm]OH.

The thermal stability of ionic liquid was tested on Labsys Evo TG-DSC (SETARAM Instrumentation, Caluire, France). Furnace atmosphere is dynamic N_2_, the oven temperature was programmed from room temperature to 500 °C at 10 °C/min, sample mass is about 5 mg. The TG spectrum of the ionic liquid is shown in Figure 2. It can be seen that ionic liquid has very high decomposition temperature. At the beginning, the ionic liquid showed a slight weight loss due to the evaporation of water. [Hnmm]OH started decomposing at 100.09 °C, and had a weight loss of 54.65% at 140.07 °C; when the temperature is above 250.90 °C, the second peak appeared, with a weight loss of 24.21% untill 278.09 °C. The good thermal stability of ionic liquid makes it promising for transesterification reaction and other catalytic reactions.

### 2.3. Effect of [Hnmm]OH Concentration

**Figure 3:**
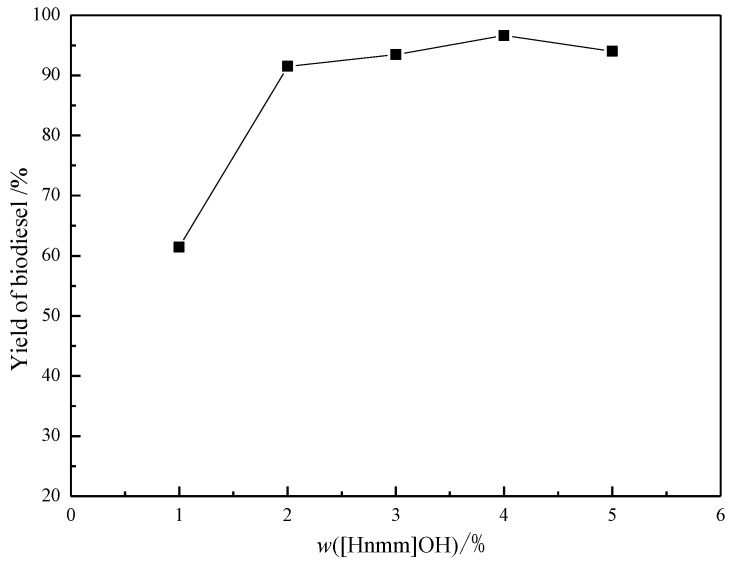
Effect of [Hnmm]OH concentration on yield of biodiesel Reaction conditions: *n*(Methanol)/*n*(Oil) = 8; *T* = 70 °C; *t* = 2 h.

The influence of catalyst concentration on the biodiesel yield is shown in Figure 3. It can be seen that the biodiesel yield was greatly increased from 61.2% to 96.6% with the increasing of catalyst dosage from 1% to 4%, when the catalyst concentration is beyond the optimum concentration 4%, the yield has no evident increase which can be ascribed to ester saponification under alkaline conditions. It should be mentioned that the reaction is very well controlled and highly reproducible.

### 2.4. Effect of the Methanol/Soybean Oil Molar Ratio

**Figure 4:**
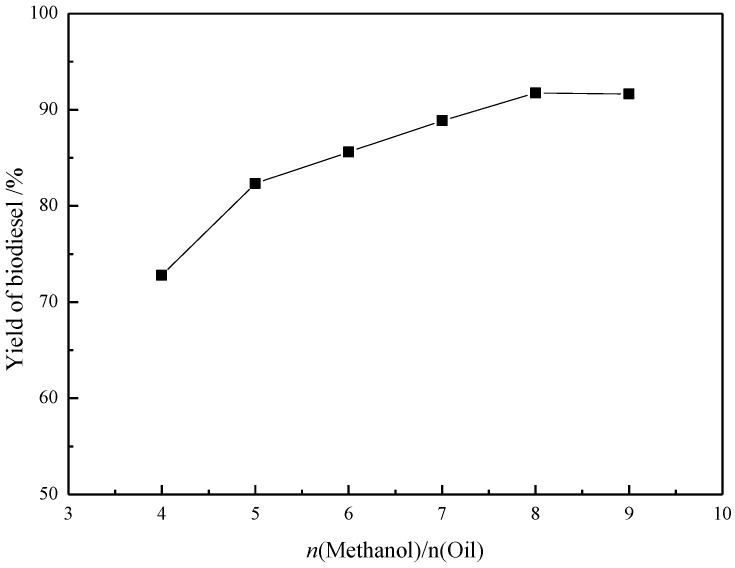
Effect of *n*(Methanol)/*n*(Oil)on the yield (y) in preparation of biodiesel catalyzed by [Hnmm]OH. Reaction conditions: *w*([Hnmm]OH = 3%); *T* = 70 °C; *t* = 2 h.

The influence of the methanol/soybean oil molar ratio on the biodiesel yield is shown in Figure 4. When the ratio of methanol/oil is increased from 4:1 to 9:1, the yield raised from 72.8% to 91.8%. The yield of biodiesel reached a maximum level at methanol/oil ratio of 8:1. Interestingly, high molar ratio leads to longer reaction time, which also makes product separation more difficult since the ILs and the product esters are soluble in methanol.

### 2.5. Effect of Reaction Temperature

**Figure 5:**
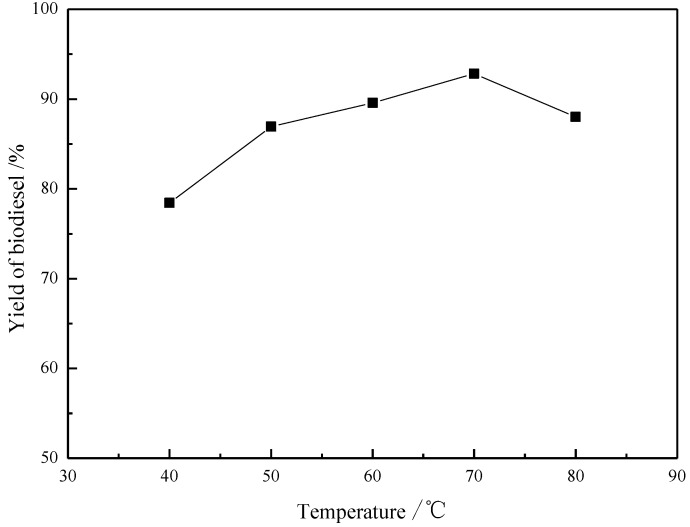
Effect of reaction temperature on the yield (y) in preparation of biodiesel catalyzed by [Hnmm]OH. Reaction conditions: *w*([Hnmm]OH = 3%); *n*(Methanol)/n(Oil) = 8; *t* = 2 h.

Figure 5 reveals the effect of reaction temperature on the biodiesel yield. The yield was increased in the temperature range from 40 to 70 °C, and reached optimum at 70 °C. Above 70 °C, the biodiesel yield was negatively affected, which is due to the methanol volatilization under high temperature. Therefore, the most appropriate temperature for the reaction is 70 °C.

### 2.6. Effect of Reaction Time

**Figure 6:**
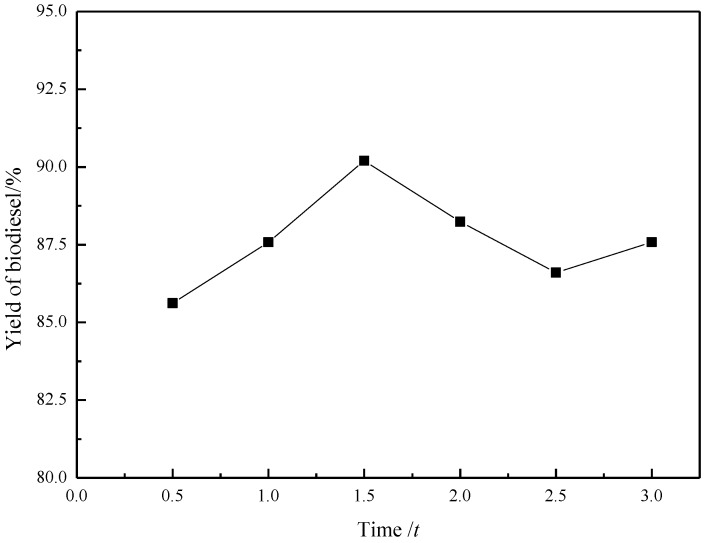
Effect of reaction time on the yield (*y*) in preparation of biodiesel catalyzed by [Hnmm]OH. Reaction conditions: *w*([Hnmm]OH = 3%); *n*(Methanol)/n(Oil) = 8; *T* = 70 °C.

The effect of the reaction time on the biodiesel yield is shown in Figure 6. The reaction time very clearly affects the yield of biotiesel, which reached highest (90%) after 1 h. After 1.5 h, the yield was decreased slightly and reached steady state. Probably, the reverse reaction of FAMEs with glycerol prevails at this stage, enabling the decrease in reaction yield.

### 2.7. Recycling of the [Hnmm]OH

**Figure 7:**
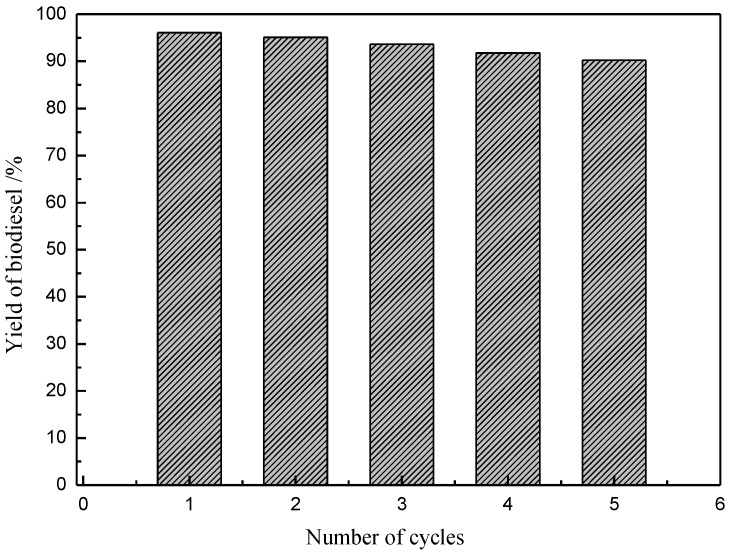
Recycling ability tests of [Hnmm]OH. Reaction conditions: *w*([Hnmm]OH) = 4 wt%, *n*(Methanol)/*n*(Oil) = 8, *T* = 70 °C, *t* = 2 h.

A great advantage of using ILs is that it is very easy to separate the final products. After the reaction, a biphasic system was obtained. The top phase contains FAMEs and a little bit of methanol. Pure FAMEs can be isolated by vacuum evacuating the methanol. The bottom phase contains methanol, glycerol and ILs. Pure glycerol can be obtained simply by distillation. After distillation, pure ILs was obtained, which can be used directly for another reaction. In order to assess the economic feasibility and sustainable development of this “green chemistry” strategy, it was necessary to investigate the recycling of IL [Hnmm]OH. Only a slight loss in activity is observed after five cycles, as shown in Figure 7. It is therefore believed that the catalyst is highly promising for further investigations.

### 2.8. Comparative Study on the Catalytic Activities in Different Catalyst

A comparative study on the catalytic activities of KOH and [Bmim]Im (1-butyl-3-methylimidazolium imidazolide) and [Hnmm]OH catalysts was carried out (Table 2). KOH showed similar activity, but it is very difficult to recycle and produce a large amount of wastewater during the production process. [Bmim]Im exhibited a high catalytic performance and a good stability for transesterification. However, the cost of materials to synthesis catalyst more higher than [Hnmm]OH, [Hnmm]OH catalyst has many advantages, such as good stability, strong basicity and less waste generation by serving as both solvent and catalyst. Moreover, comparing with conventional catalysts such as KOH and NaOH, ionic liquid catalyst is much less sensitive to water and free fatty acid.

**Table 2:** Comparison of catalysis with different catalysts.

Catalyst	Yield of biodiesel/%
KOH	97.8
[Hnmm]OH	97.0
[Bmim]Im [16]	95

### 2.9. Comparison of the Properties of the as Prepared Biodiesel with Specifications

The properties of the as prepared biodiesel were studied in great details. As can be seen from Table 3, the as prepared biodiesel has great properties, all of which are within the specifications.

**Table 3:** Comparison of the properties of the as prepared biodiesel with specifications.

Properties	Biodiesel	Specification	Standard method
density (20 °C)/(kg/m^3^)	875.2	820–900	GB/T2540
viscosity (40 °C)/(mm^2^/s)	4.36	1.9–6.0	GB/T265
flash point (close cup)/°C	171	>130	GB/T261
cold filter pour point/°C	3	report	SH/T0248
pour point/°C	0	report	SH/T0248
sulfur content/(mass)/%	0.0078	≤0.05	SH/T0689
Carbon res idual(mass)/%	0.25	≤0.3	GB/T17144
ash content/%(mass)	0.01	≤0.020	GB/T2433
water content/(Vol)/%	trace	≤0.05	SH/T0246
Cu Corrosion(50 °C, 3 h)/Grade	1	≤1	GB/T5096
centane number	51	≥49	GB/T386
oxidative stability(110 °C)/h	6.5	≥6.0	EN14112
acidity value/mg KOH (g·oil)^−1^	0.48	≤0.80	GB/T264
Free Glycerol content (mass)/%	0.015	≤0.02	ASTMD6584
Total Glycerol content(mass)/%	0.21	≤0.25	ASTMD6584
distillation range (90%)/°C	348	≤360	GB/T6536

## 3. Experimental Section

### 3.1. Materials

Materials: *N*-methyl morpholine, Morpholine, 1-butyl bromide, potassium hydroxide, Anhydrous methanol, Ethanol, Ethyl acetate, and Anhydrous ether were used (grade of analytical reagent). Soybean oil was obtained from the local market.

### 3.2. Preparation of the Alkaline Ionic Liquid [Hnmm]OH

The alkaline ionic liquid [Hnmm]OH was synthesized in the following way: a certain amount of distilled *N*-methyl morpholine was poured into a 250 mL flask containing a constant pressure funnel, a reflux condenser, magnetic stirrer and a gas inlet. The flask was placed in a 70 °C water bath and rapidly stirred under N_2_ atmosphere. 1-Butyl bromide was added dropwise, and the water bath temperature was maintained at 70 °C and vigorous stirring for 12 h, the molar ratio of *N*-methyl morpholine and 1-butyl bromide was 1:1.1. White crystals crude product 1-butyl-3-methyl morpholine bromide ([Hnmm]Br) was obtained. The mixture was then cooled down to room temperature and filtered to give a solid, which was washed repeatedly with small portions of hot ethyl acetate and vacuum dry evaporated at 80 °C for 5 h under vacuum.

**Figure 8:**
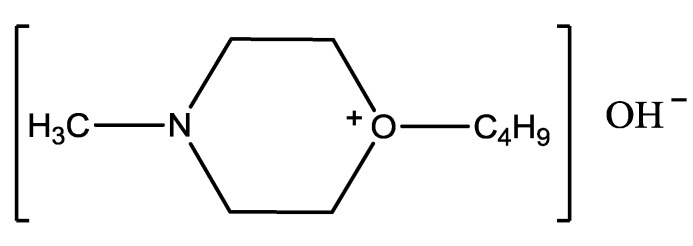
Chemical structure of alkaline ionic liquid [Hnmm]OH.

Equimolar [Hnmm]Br was added to a solution of KOH in MeOH, and the misxture was vigorously stired at room temperature for 24 h. After the reaction, the precipitated KBr was filtered off, and the filtrate was evaporated to leave crude [Hnmm]OH as a viscous liquid. After removal of solvent, in order to prepare the pure ionic liquid for use, the crude product was extracted with Et_2_O for three times and vacuum dry evaporated at 70 °C for 10 h under vacuum [23,24,25]. Structure of alkaline ionic liquid [Hnmm]OH prepared was shown in Figure 8.

[Hnmm]Br: ^1^H-NMR (500 MHz, d-CDCl_3_), 1.02 (*t*, 3H, CH_3_), 1.454–1.529 (*m*, 2H, CH_2_), 1.771–1.819 (*m*, 2H, CH_2_), 3.593 (*s*, 3H, CH_3_), 3.623–3.673 (*m*, 2H, CH_2_), 3.855–3.904 (*t*, 4H, CH_2_), 4.017–4.134 (*m*, 4H, CH_2_).

[Hnmm]OH: ^1^H-NMR (500 MHz, d-CDCl_3_), 0.94–0.97 (*t*, 3H, CH_3_), 1.39–1.45 (*m*, 2H, CH_2_), 1.71–1.75 (*m*, 2H, CH_2_), 3.22 (*s*, 2H, CH_2_), 3.28 (*s*, 3H, CH_3_), 3.37 (*s*, 4H, CH_2_), 3.71–3.98 (*m*, 4H, CH_2_). Anal. calcd: C, 61.67; H, 12.08; N, 7.99. Found: C, 61.37; H, 11.82; N, 7.66.

### 3.3. Synthesis of Biodiesel

**Scheme 1:**
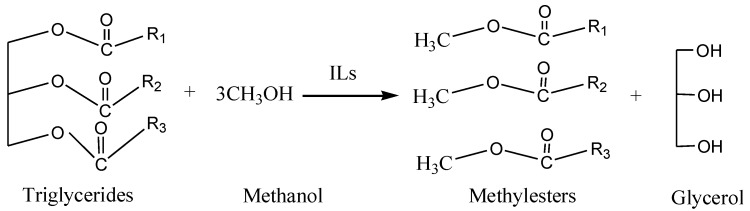
The synthesis of the biodiesel.

A certain amount of soybean oil was charged into a 250 mL flask equipped with a reflux condenser, magnetic stirrer. The flask was placed in a water bath at reaction temperature, then a certain amount of a solution of [Hnmm]OH in MeOH was added. After the reaction products had settled, the upper layer mainly consisting of biodiesel was separated and washed with hot distilled water to remove the impurities such as soap, unreacted methanol and residual ionic liquid. The washed crude biodiesel was dried under vacuum at 90 °C for 5 h. the process was illustrated in Scheme 1.

### 3.4. Analytical Methods

#### 3.4.1. Preliminary Physical and Chemical Properties of Soybean Oil

Acid value (*AV*) (mg KOH/g) was calculated as follows:

(1) $$AV=\frac{(V_{1}-V_{0})\times C\times 56.1}{m}\times 100\%$$

where *V_0_* is initial volume of the KOH; *V_1_* is final volume of KOH; *C* is the concentration of KOH; 56.1 is molar mass of KOH; *m* is the mass of soybean oil;

The saponification number (*SV*) was calculated as follows:

(2) $$SV=\frac{(V_{2}-V_{1})\times c\times 56.1}{m}$$

where *V_1_* is the volume of HCl titrating sample, *mL*; *V_2_* is the volume of HCl titrating blank sample, mL; *c* is the concentration of HCl, mol/L; *m* is the mass of sample, g; 56.1 is molar mass of KOH g/mol.

The average molecular weight of soybean oil (*M*) was calculated as follows:

(3) $$M=\frac{56.11\times 1000\times 3}{\left(SV-AV\right)}$$

#### 3.4.2. The Determination of the Yield of Biodiesel

The biodiesel products were analyzed by GC-1690 using a flame ionization detector (FID) and small caliber capillary column of SE-30. The oven program was set to an initial temperature of 100 °C (held for 10 min), increasing to 230 °C at a rate of 20 °C·min^−1^, where the temperature was held for 10 min. The temperatures of injector and interface were both held constant at 260 °C. The internal standard method for determinating the yield of biodiesel was used, with diethyl adipate as internal standard. The yield of biodiesel was calculated using the following equation:

(4) $$BDF\%=\frac{\frac{\text{Ai1}}{\text{As1}}\times \frac{\text{mi}1}{\text{W}1}}{\frac{\text{Ai2}}{\text{As2}}\times \frac{\text{mi}2}{\text{W}2}}\times 100\%$$

where BDF% is the yield of biodiesel; *Ai1* is the peak area of analytes; *Ai2* is the peak area of sample; *As1* or *As2* is the peak area of internal standard; *mi*1 is the mass of analytes, *g*(accurate to 0.0001 *g*); *W*1 or *W*2 is the mass of internal standard, *g* (accurate to 0.0001 *g*).

#### 3.4.3.The Structure Analysis of Ionic Liquids

FT-IR was performed on a Nicolet PROTÉGÉ 460 Fourier transform infrared spectroscopy (Thermo Scientific, Waltham, MA, USA), KBr pellet liquid membrane.

The ^1^H-NMR measurements were carried out on a AVANCEIII 500 MHz spectrometer (Bruker, Billerica, MA, USA) using CDCl3 as solvent, magnetic intensity is 11.75 T, resolution < 0.2 Hz, sensitivity > 300 (1 H).

## 4. Conclusions

The IL [Hnmm]OH was synthesized easily by a two-step method, which was characterized by ^1^H NMR and FT-IR. This IL [Hnmm]OH showed high basicity and good thermal stability. The catalyst had an excellent catalytic performance at 70 °C reaction temperature, 1.5 h of reaction time, 4 wt% catalyst dosage and n(Methanol)/n(Oil) = 8:1. The recycling test showed that the catalyst could be easily isolated from the reaction mixture and used five times without much loss in catalytic activity.
